# A quantitative feeding assay in adult *Drosophila* reveals rapid modulation of food ingestion by its nutritional value

**DOI:** 10.1186/s13041-015-0179-x

**Published:** 2015-12-21

**Authors:** Wei Qi, Zhe Yang, Ziao Lin, Jin-Yong Park, Greg S. B. Suh, Liming Wang

**Affiliations:** Life Sciences Institute, Zhejiang University, Hangzhou, Zhejiang 310058 China; Innovation Center for Cell Signaling Network, Zhejiang University, Hangzhou, Zhejiang 310058 China; Department of Cell Biology, Skirball Institute of Biomolecular Medicine, Neuroscience Institute, New York University School of Medicine, New York, NY USA

**Keywords:** Starvation, Nutritive sugar, Feeding, *Drosophila*

## Abstract

**Background:**

Food intake of the adult fruit fly *Drosophila melanogaster*, an intermittent feeder, is attributed to several behavioral elements including foraging, feeding initiation and termination, and food ingestion. Despite the development of various feeding assays in fruit flies, how each of these behavioral elements, particularly food ingestion, is regulated remains largely uncharacterized.

**Results:**

To this end, we have developed a manual feeding (MAFE) assay that specifically measures food ingestion of an individual fly completely independent of the other behavioral elements. This assay reliably recapitulates the effects of known feeding modulators, and offers temporal resolution in the scale of seconds. Using this assay, we find that fruit flies can rapidly assess the nutritional value of sugars within 20–30 s, and increase the ingestion of nutritive sugars after prolonged periods of starvation. Two candidate nutrient sensors, SLC5A11 and Gr43a, are required for discriminating the nutritive sugars, D-glucose and D-fructose, from their non-nutritive enantiomers, respectively. This suggests that differential sensing mechanisms play a key role in determining food nutritional value.

**Conclusions:**

Taken together, our MAFE assay offers a platform to specifically examine the regulation of food ingestion with excellent temporal resolution, and identifies a fast-acting neural mechanism that assesses food nutritional value and modulates food intake.

**Electronic supplementary material:**

The online version of this article (doi:10.1186/s13041-015-0179-x) contains supplementary material, which is available to authorized users.

## Background

**Fig. 1:**
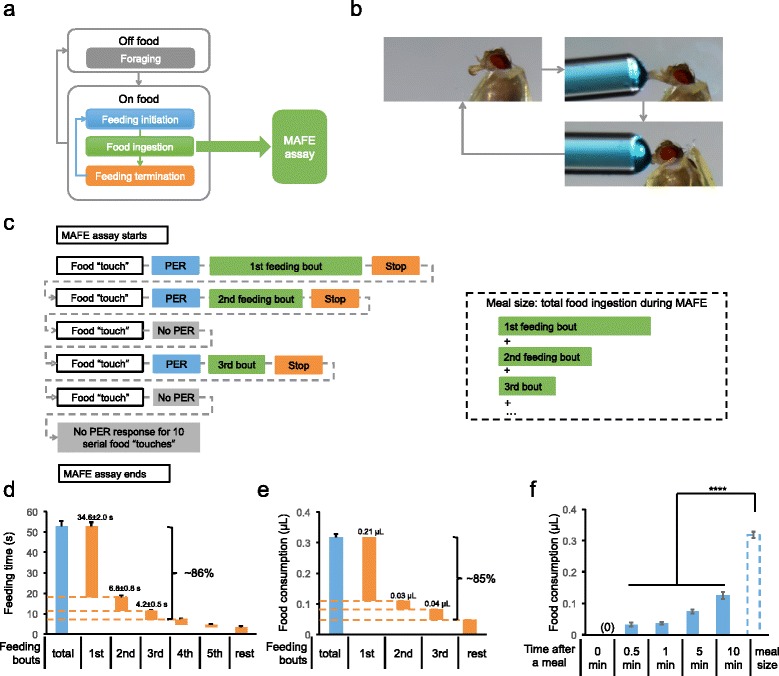
The MAFE assay to quantify food ingestion. **a** In adult fruit flies, the total food intake is determined by multiple interconnected behavioral elements including foraging (grey), feeding initiation (blue), feeding termination (orange), and food ingestion (green). We here developed a quantitative MAFE assay to specifically measure food ingestion (green). **b** A representative illustration of the MAFE assay. **c** A diagram illustrating the protocols of the MAFE assay. For details, refer to Material and Methods. **d** A waterfall chart showing the cumulative duration of feeding bouts during a meal on 100 mM sucrose (*n* = 64). The blue bar represents the total duration of a meal. The height between the upper and lower bound of each orange bar represents the duration of an indicated feeding bout(s). **e** A waterfall chart showing the cumulative ingested volume of feeding bouts during a meal on 100 mM sucrose (*n* = 29-102). The blue bar represents the total ingested volume of a meal. The height of the upper and lower bound of each orange bar represents the ingested volume of an indicated feeding bout(s). **f** Volume of 100 mM sucrose consumed by flies at different time points after the previous meal (*n* = 22-102). The “meal size” bar is re-plotted from Fig. 1d illustrating the total volume ingested during a meal. Sample size for each data set was summarized in Tab. S1. Error bars represent SEM. ns, *P* > 0.05; *P < 0.05; **P < 0.01; ***P < 0.001; ****P < 0.0001. One-way ANOVA followed by Bonferroni *post hoc* test was used for comparisons for more than 2 groups. Two-way ANOVA (and *post hoc* test if applicable) was applied for comparisons with more than one variant

Energy homeostasis of all animal species relies on a precise balance between energy intake and expenditure. For intermittent feeders including humans, feeding behavior usually begins with off-food foraging, followed by one or multiple rounds of feeding initiation, food ingestion, and feeding termination before leaving food source and starting a new round of foraging (Fig. 1a) [1]. Although the neural control of food intake has been extensively studied in rodent and insect model organisms [2], it is often unclear whether the modulation of food intake occurs through changes in foraging, feeding initiation and termination, food ingestion, or combinations of these elements. To better understand the neural regulation of energy homeostasis, it is critical to investigate how these behavioral elements of food intake are independently regulated.

A wealth of behavioral assays have been developed to quantify different aspects of feeding behavior in the adult fruit fly *Drosophila melanogaster*, which is also an intermittent feeder [3]. Starvation-induced hyperactivity and odor-driven food search have been used as measurements of foraging activity in flies [4, 5]. Two recently developed automated assays, named FLIC (Fly Liquid-Food Interaction Counter) and flyPAD (fly Proboscis and Activity Detector), quantify the physical contact of flies’ proboscis and food surface through monitoring the changes in conductance and capacitance, respectively [6, 7]. These assays therefore offer quantitative measurements of feeding initiation, such as the frequency of the proboscis extension reflex (PER) responses. However, a behavioral assay that specifically measures food ingestion without influence of the other behavioral elements of food intake still lacks. Labeling food with radioactive or colored substrates has been used to measure food ingestion of a group of flies [8, 9]. These methods, however, only offer semi-quantitative measurements of food ingestion and preference, and lack the resolution at individual flies. The capillary feeder (CAFE) assay continuously and reliably detects the volume of liquid food being ingested [10]. But foraging behavior becomes a major confound because the CAFE setup requires flies to reliably locate food source at a tiny tip of a hanging capillary [3], a behavior task that is not natural to flies and requires foraging capability. This assay also requires a longer time course (i.e. hours to days) [3].

We have herein developed a novel and quantitative feeding assay, named MAFE assay, to address the caveats associated with previous feeding assays. The MAFE assay quantifies food ingestion without the interference from foraging and feeding initiation, and offers temporal resolution in the scale of seconds. We find that the MAFE assay reliably recapitulates the effects of known feeding modulators including food deprivation and gut stretch. Using this assay, we further uncover that flies can detect the nutritional value of D-glucose and D-fructose, the two dietary sugars for fruit flies, and increase food ingestion after prolonged periods of starvation [11, 12]. Intriguingly, the discrimination of these nutritive sugars from their non-nutritive enantiomers only takes 20–30 s, and requires two candidate nutrient sensors, SLC5A11 and Gr43a, respectively [12, 13]. This suggests that a fast-acting mechanism plays a critical role in determining the nutritional value of sugars in *Drosophila*.

## Results and discussion

### The development of the MAFE assay

We developed a quantitative MAFE assay to specifically measure food ingestion in an adult fly (Fig. 1a-c). An individual fly was immobilized in a 200 μL pipette tip (Fig. 1b, *left*), and presented with liquid food filled in a graduated glass capillary. In successful feeding events, flies started feeding by extending their proboscis into the food (Fig. 1b, *middle*). After we confirmed that flies had normal PER responses to food, we fed the flies until they stopped responding to a series of ten food stimuli (Fig. 1b, *right*). The total volume of liquid food consumed between each round of feeding initiation and termination would be a measurement of food ingestion (Fig. 1c). Hence, the MAFE assay specifically measures food ingestion independently from potential changes in foraging behavior. As PER and food consumption can be separately assayed in the MAFE assay, it can also separate the effects on food ingestion from those on feeding initiation (for example, see Fig. 3a-b).

**Fig. 2:**
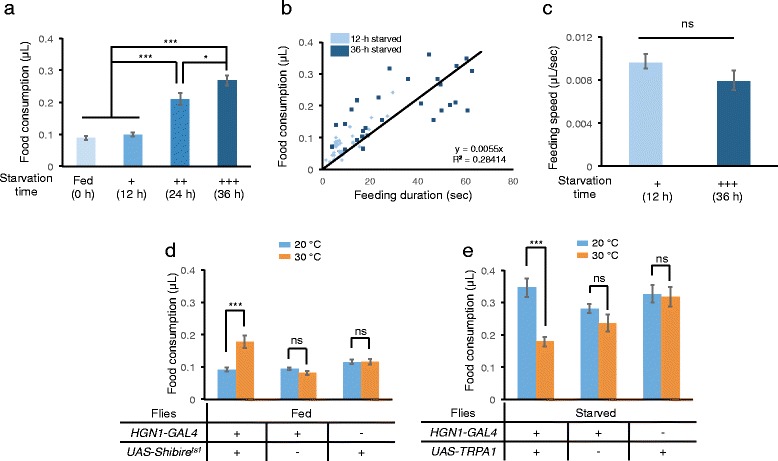
The MAFE assay detects the effects of food deprivation and gut stretch on food ingestion. **a** Volume of 100 mM sucrose consumed in a meal, by fed *Canton-S* flies, and those starved for 12, 24 and 36 h (*n* = 25-29). **b** Duration (x-axis) and volume of 100 mM sucrose consumed (y-axis) in the first feeding bout by *Canton-S* flies starved for 12 and 36 h (*n* = 29-34). The regressed linear equation and the R^2^ value are listed in the plot. **c** Feeding speed of *Canton-S* flies starved for 12 and 36 h (*n* = 29-34). **d** Volume of 100 mM D-glucose consumed in a meal, by fed flies with indicated genotypes at 20 °C or 30 °C (*n* = 24-39). **e** Volume of 100 mM D-glucose consumed in a meal, by starved (24–36 h) flies with indicated genotypes at 20 °C or 30 °C (*n* = 19-35). Sample size for each data set was summarized in Tab. S1. Error bars represent SEM. ns, *P* > 0.05; *P < 0.05; **P < 0.01; ***P < 0.001; ****P < 0.0001. One-way ANOVA followed by Bonferroni *post hoc* test was used for comparisons for more than 2 groups. Two-way ANOVA (and *post hoc* test if applicable) was applied for comparisons with more than one variant

Having established this assay, we next determined the kinetics of food ingestion revealed by the MAFE assay. Flies starved for 36 h usually exhibited several bouts of feeding behavior before they stopped responding to food stimuli. The duration and ingested volume of each feeding bout was recorded. As shown in Fig. 1d, 36-h starved flies spent 52.8 ± 2.6 s on average in feeding when presented with sucrose. The first feeding bout took 34.6 ± 2.0 s, and the first three feeding bouts together made up to ~86 % of the total feeding duration (Fig. 1d). The starved flies ingested 0.32 ± 0.01 μL liquid food on average, and the first three feeding bouts together comprised up to ~85 % of the total feeding volume (Fig. 1e). These flies ingested significantly smaller volumes of food when presented with sucrose again in the next 10 min after the MAFE assay (Fig. 1f), suggesting that they remained satiated and uninterested in food for the following short period. These results indicate that the total volume of food ingestion during the course of our MAFE assay likely defines the size of a “meal” (Fig. 1c, *right*), and therefore can be used as a measurement to investigate the regulation of food ingestion. It is also worth noting that the meal size of fed flies in the MAFE assay (Fig. 2a) is similar to that in the CAFE assay [10], suggesting that food ingestion of immobilized flies is comparable to that of freely moving flies.

### The MAFE assay recapitulates the effect of food deprivation and gut stretch on food ingestion

We asked whether the MAFE assay could reliably recapitulate the effect on food ingestion by known modulators of feeding behavior. Food deprivation promotes feeding behavior (also see Additional file 1: Figure S1a-b) [6, 14]. Consistently, we found that the length of starvation significantly increased food ingestion, from 0.10 ± 0.01 μL in fed flies to 0.21 ± 0.02 μL in 24-h starved flies and 0.27 ± 0.02 μL in 36-h starved flies (Fig. 2a).

We further asked how starvation increased food consumption: whether it promoted feeding speed or feeding duration, or both. To this end, we assayed the duration and ingested volume from the first feeding bout of 12-h and 36-h starved flies. As shown in Fig. 2b, the ingested volume for each fly showed a modest correlation with the duration of the feeding bout. We also found that the feeding speed was not significantly changed by a longer period of starvation (Fig. 2c). Taken together, these results suggest that starvation enhances food ingestion via increased duration of feeding rather than increased speed.

Gut stretch has also been shown to suppress the total food intake over a 24-h period by the activation of HGN1^+^ mechanosensory neurons innervating the gut [15]. Consistent with this, acute silencing of HGN1^+^ neurons by ectopically expressing a temperature sensitive form of dynamin protein (Shibire^ts1^) increased food ingestion in fed flies at a restrictive temperature (Fig. 2d), while acute activation of these neurons by ectopically expressing a temperature sensitive TRP channel (TRPA1) reduced food ingestion in starved flies (Fig. 2e) [16, 17]. In conclusion, our MAFE assay reliably quantifies food ingestion of individual flies without a potential confound from other behavioral elements that also influence feeding behavior.

### Rapid detection of nutritive sugars by flies after prolonged periods of starvation

Recent reports have shown that fruit flies can detect the nutritional value of sugars and modulate food preference through a mechanism independent of peripheral gustatory perception (also see Additional file 1: Figure S1c-d) [11, 18]. Because food preference was measured over the course of several hours in their assays, however, it was difficult to determine how fast flies can detect the caloric value of sugars and therefore, the underlying neurobiological and metabolic mechanisms. Because our MAFE assay offers a good temporal resolution (Fig. 1d), we sought to examine the effect of food nutritional value on food ingestion by using this assay.

**Fig. 3:**
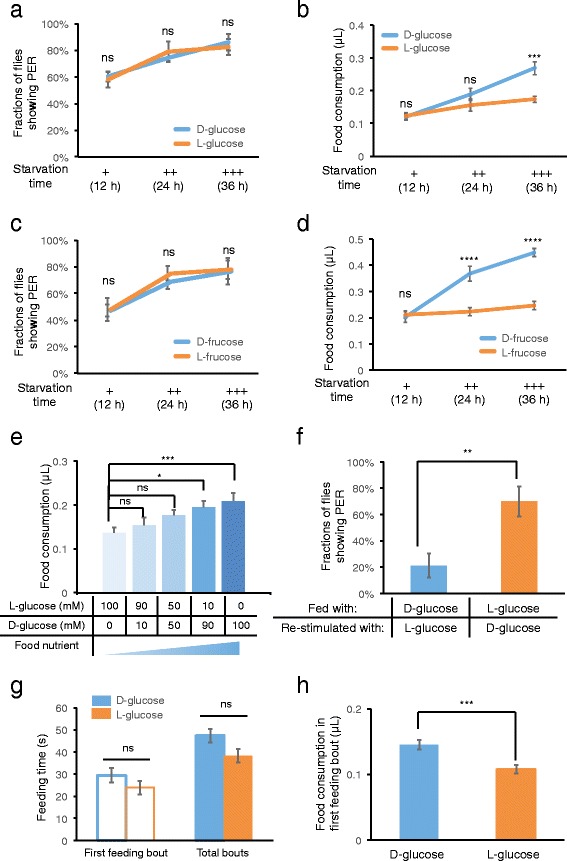
Starved flies can assess food nutritional value and modulate food ingestion. **a, c** Fractions of *Canton-S* flies exhibiting PER responses to (**a**) 100 mM D-/L-glucose and (**c**) 100 mM D-/L-fructose when starved for 12, 24, and 36 h (*n* = 19-36). **b, d** Volume of (**b**) 100 mM D-/L-glucose and (**d**) 100 mM D-/L-fructose consumed in a meal, by *Canton-S* flies starved for 12, 24 and 36 h (*n* = 19-36). **e** Volume of sugar solutions with indicated D- and L-glucose concentrations consumed in a meal, by 36-h starved *Canton-S* flies (*n* = 22-35). **f** Fractions of 36-h starved *Canton-S* flies exhibiting PER responses to the indicated second sugar (“Re-stimulated with”) after finishing a meal with the indicated first sugar (“Fed with”) (*n* = 25-26). **g** Feeding time of 36-h starved *Canton-S* flies when fed with 100 mM D-/L-glucose (*n* = 24-30). **h** Volume of 100 mM D-/L-glucose consumed during the first feeding bout of a meal by 36-h starved *Canton-S* (*n* = 68-69). Sample size for each data set was summarized in Tab. S1. Error bars represent SEM. ns, *P* > 0.05; *P < 0.05; **P < 0.01; ***P < 0.001; ****P < 0.0001. Student’s *t*-test was used for pair wise comparisons. One-way ANOVA followed by Bonferroni *post hoc* test was used for comparisons for more than 2 groups. Two-way ANOVA (and *post hoc* test if applicable) was applied for comparisons with more than one variant

D-glucose is one of the major carbohydrate sources for fruit flies [19]. L-glucose, its enantiomer, cannot be metabolized by flies and therefore, provides no nutritive value [20]. Nonetheless, both molecules evoked strong and comparable PER responses in starved flies, confirming that these two sugars are equally palatable to initiate feeding behavior (Fig. 3a) [11, 20]. We asked whether flies could respond differently to these two enantiomers during the course of a single meal. Using the MAFE assay, we found that 36-h starved flies ingested more than 50 % volume on D-glucose than on L-glucose (0.27 ± 0.02 μL for D-glucose and 0.17 ± 0.01 μL for L-glucose), whereas flies starved for shorter time periods (12 and 24 h) exhibited no difference (Fig. 3b). Similar effects were also observed for higher concentrations of D-/L-glucose (Additional file 1: Figure S2). Besides D-glucose, D-fructose is the other dietary sugar for flies [19]. Similar to D- and L-glucose, both D- and L-fructose enantiomers elicited comparable PER responses in starved flies (Fig. 3c). However, flies starved for 24 and 36 h consumed greater volumes of D-fructose than those of L-fructose (Fig. 3d).

Taken together, flies can detect the nutritional value of sugars, and enhance food ingestion of nutritive sugars upon food deprivation. Consistent with this hypothesis, increasing nutritional content of sugar enhanced food ingestion of 36-h starved flies in a dose-dependent manner (Fig. 3e). Most of the 36-h starved flies pre-fed with L-glucose still responded to subsequent re-stimulation of D-glucose, while starved flies pre-fed with D-glucose failed to respond to L-glucose, confirming that starved flies could be sated by nutritive D-glucose but not by non-nutritive L-glucose (Fig. 3f).

Intriguingly, starved flies could detect the nutritional value of sugars during the course of a single meal, which took 47.4 ± 5.3 s on average for D-glucose and 38.2 ± 3.1 s for L-glucose (Fig. 3g, “Total bouts”). We further found that 36-h starved flies consumed greater volumes of D-glucose than those of L-glucose during the first feeding bout of a meal (Fig. 3h, 0.14 ± 0.01 μL for D-glucose vs. 0.11 ± 0.01 μL for L-glucose); and it only took 29.5 ± 3.3 s for D-glucose and 23.9 ± 3.1 s for L-glucose (Fig. 3g, “First feeding bout”). These results highlight the presence of a fast-acting mechanism that detects food nutritional value and modulate food ingestion within 20–30 s. Our data are also consistent with the hypothesis that flies are capable of directly detecting nutritive sugar through a mechanism medicated by a small group of neuropeptidergic neurons in the fly brain [21].

### SLC5A11 and Gr43a mediates the discrimination of nutritive vs. non-nutritive sugars

**Fig. 4:**
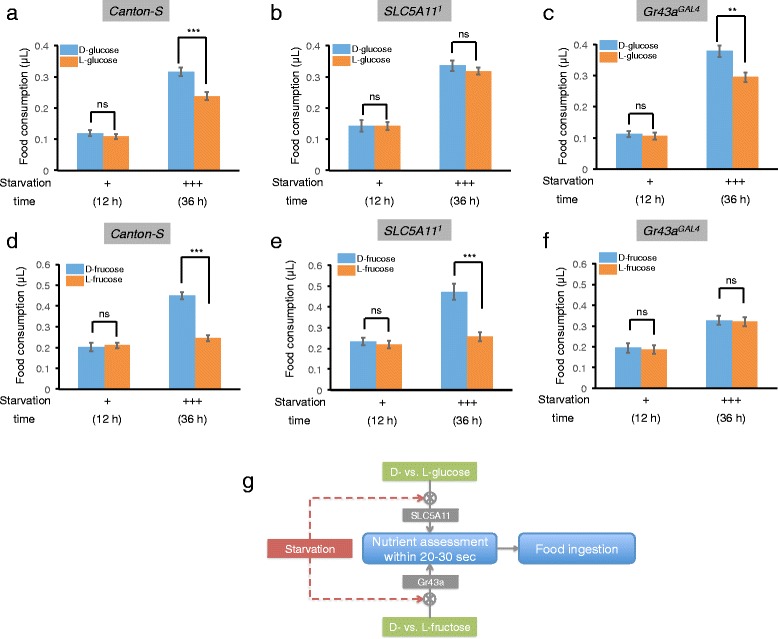
Differential sensing mechanism of nutritive sugars. **a**-**c** Volume of 100 mM D-/L-glucose consumed in a meal, by flies with indicated genotypes starved for 12 and 36 h (*n* = 24-46). **d**-**f** Volume of 100 mM D-/L-fructose consumed in a meal, by flies with indicated genotypes starved for 12 and 36 h (*n* = 19-36). **g** A working model. Starved flies can assess the nutritional value of food (i.e. the presence of D- vs. L-glucose and D- vs. L-fructose) within 20–30 s and modulate food ingestion accordingly. SLC5A11 and Gr43a are required for the food nutrient assessment, respectively. Sample size for each data set was summarized in Tab. S1. Error bars represent SEM. ns, *P* > 0.05; *P < 0.05; **P < 0.01; ***P < 0.001; ****P < 0.0001. Student’s *t*-test was used for pair wise comparisons. One-way ANOVA followed by Bonferroni *post hoc* test was used for comparisons for more than 2 groups. Two-way ANOVA (and *post hoc* test if applicable) was applied for comparisons with more than one variant

To further investigate the mechanism underlying the increased food ingestion of nutritive sugars, we examined two recently identified candidate food nutrient sensors, SLC5A11 and Gr43a, in our MAFE assay [12, 13]. SLC5A11, a brain-expressing Na^+^/solute co-transporter, is required for taste-independent nutrient selection in starved flies [13]. It was not clear, however, whether it was involved in the regulation of food ingestion *per se*. Unlike *Canton-S* controls (Fig. 4a), *SLC5A11*^*1*^ hypomorphic mutant flies ingested comparable food volumes of D-glucose as those of L-glucose even after 36-h of starvation (Fig. 4b). This result indicates that SLC5A11 is required for discriminating nutritive D-glucose from non-nutritive L-glucose after starvation.

Moreover, starved *Canton-S* flies, but not *SLC5A11*^*1*^ mutants, exhibited a preference towards an odorant associated with nutritive D-glucose over another odorant associated with L-glucose in an appetitive olfactory conditioning assay. This suggests that starved flies are able to sense the difference between D-glucose and L-glucose in an SLC5A11 dependent manner (Additional file 1: Figure S3). Consistent with the observation using the MAFE assay, flies can quickly associate the nutritional content of sugar with an odorant within 2 min. It remains unclear, however, whether SLC5A11 is involved in directly sensing the nutritional value of glucose, or regulating food ingestion. Gr43a, a brain-expressing gustatory receptor, was implied in the detection of hemolymph nutrient and the regulation of feeding behavior [12]. *Gr43*^*GAL4*^ mutant flies were still capable of discriminating D- vs. L-glucose when starved (Fig. 4c). Interestingly, Gr43a, but not SLC5A11, was required for detecting the nutritional value associated with D-fructose (Fig. 4d-f). These results highlight the difference in the mechanisms by which these molecules mediate behavior responses to different sugars (Fig. 4g).

In summary, we have developed a quantitative MAFE assay to investigate the regulation of food ingestion without the interference from other feeding related behavioral elements. Using this assay, we found that fruit flies could assess food nutritional value within 20–30 s, and enhance food ingestion of nutritive sugars after prolonged starvation. Notably, the detection of D-glucose and D-fructose, two major dietary sugars for fruit flies in natural habitats, employs distinct neural mechanisms involving two candidate nutrient sensors SLC5A11 and Gr43a, respectively. Collectively, our study offers a starting point to further investigate how the food nutritional value influences different behavioral elements of feeding.

## Conclusions

Here we describe a novel and quantitative feeding assay to examine the regulation of food ingestion precisely during the first bouts of feeding. Using the assay, we find that flies could detect the nutritional value of sugars, D-glucose and D-fructose, within 20–30 s and enhance food ingestion after prolonged starvation. Two candidate nutrient sensors SLC5A11 and Gr43a are required for discriminating the nutritive sugars from their non-nutritive enantiomers. Taken together, we have developed a new assay with good behavioral specificity and temporal resolution to investigate food ingestion and identify a fast-acting mechanism that senses the nutritional value of sugar.

## Methods

### Flies

Flies were kept in vials containing standard fly medium made of yeast, corn, and agar at 25 °C and 60 % humidity and on a 12-h light:12-h dark. *Canton-S* virgin female flies aged for 4–6 days were used for all behavioral experiments unless otherwise indicated. *SLC5A11*^*1*^ (*y*^*1*^*w*^*67c23*^*; P{EPgy2}SLC5A11*^*EY21708*^) mutant (hypomorphic allele) was from Bloomington (#22498) [13]. *Gr43a*^*GAL4*^ mutant (null allele) was from Hubert Amrein (Texas A&M Health Science Center) [12]. *HGN1-GAL4* was from Zhiqiang Yan [22]. *UAS-Shibire*^*ts1*^ was from Gerald M. Rubin [17]. UAS-TRPA1 was from Paul A. Garrity [16].

### Chemicals

Agar (A1296) and sucrose (S7903) were purchased from Sigma-Aldrich. D-(−)-Fructose (F0060), L-(+)-Fructose (F0317), D-(+)-Glucose (G0048), and L-(−)-Glucose (G0226) were purchased from TCI (Tokyo Chemical Industry).

### MAFE assay

Flies were fed as described previously [4]. Briefly (as illustrated in Fig. 1a-c), 4–6 days old virgin female flies were fed or wet starved in vials containing 5 % sucrose plus 2 % agar, or 2 % agar only, before the assay, respectively. Individual flies were then gently aspirated into a 200 μL pipette tip and satiated with water stimulation delivered by a pipette tip. Subsequently, 3 μL liquid food (added with 5 % blue dye from McCormick) in a fine graduated capillary (VWR, #53432-604) was delivered to the proboscis of flies. As illustrated in Fig. 1a-c, if a feeding event occurred, the flies would fully extend proboscis and start drinking the liquid food. The tip of the capillary could be retrieved a bit away from the flies to allow the full extension of proboscis. Once the flies stopped feeding and retrieved proboscis, the food stimulation was repeated until the flies became unresponsive to a series of 10 food stimuli. Flies that exhibited prolonged water consumption or no PER to liquid food were excluded from the calculation of average meal size.

Before the MAFE assay, various amounts of liquid (1 μL, 2 μL, and 3 μL) were injected into each capillary for fine calibration (i.e. the linear correlation between the length of the liquid column in the capillary and its volume). Before and after the MAFE assay for each fly, the length of the liquid column was carefully measured by an electronic vernier caliper and was used to calculate the ingested volume by a single fly. The accuracy of the assay could reach 0.01 μL scale.

To quantify the duration of food ingestion, two experimenters were required, with one conducting the MAFE assay while the other recording the start and stop point for each feeding bout by using a stop watch or timer.

PER. PER was assayed as described previously [4, 23]. Briefly, flies were prepared and water satiated as in the MAFE assay and subjected to different sugar solutions. Each sugar solution was presented twice, and flies showing PER to at least one of the two trials were considered positive to that concentration.

## Additional file

Additional file 1:
**Supplementary methods.** **Figure S1.** Starvation and food nutrient content promote feeding behavior from long-term FLIC assays. **Figure S2.** Starved flies also exhibited preference towards nutritive D-glucose at high concentrations. **Figure S3.** SLC5A11 is required for associative learning between a nutritious sugar and an odorant in a short time window. **Table S1.** Sample size for each data set. (PDF 1197 kb)
