# Supplementary material for: A quantitative feeding assay in adult Drosophila reveals rapid modulation of food ingestion by its nutritional value
Source: Mol Brain. 2015 Dec 21;8:87. doi: 10.1186/s13041-015-0179-x (PMC4687088; doi:10.1186/s13041-015-0179-x)
Supplement: Additional file 1: — Supplementary methods. Figure S1. Starvation and food nutrient content promote feeding behavior from long-term FLIC assays. Figure S2. Starved flies also exhibited preference towards nutritive D-glucose at high concentrations. Figure S3. SLC5A11 is required for associative learning between a nutritious sugar and an odorant in a short time window. Table S1. Sample size for each data set. (PDF 1197 kb) [file 13041_2015_179_MOESM1_ESM.pdf]

SUPPLEMENTARY INFORMATION

Supplementary methods

FLIC. FLIC (Fly Liquid Interaction Counter) assays were performed as described previously (1). Briefly, one or more *Drosophila* Feeding Monitors (DFM) was connected to computer via Master Control Unit (MCU). Each DFM could test 12 flies for single-choice feeding assays or 6 flies for two-choice feeding assays. For single-choice feeding assay (Fig. S1a-b), both feeding channels were filled with 100 mM D-glucose. Flies starved for 12, 24, or 36 hours were introduced into feeding arenas by gentle aspiration, and their feeding activity was recorded for 1 hour. FLIC measured the electrical current through flies during their physical contact with liquid food. According to the original report, a.u. larger than 120 was considered as actual feeding event (red line) and the total feeding time was calculated accordingly. For two-choice feeding assay (Fig. S1c-d), two feeding channels were filled with 100 mM D- and L-glucose, respectively.

Appetitive Olfactory Conditioning Assay. Appetitive olfactory conditioning assay was performed as described previously (2). Briefly, *Canton-S* male flies were first food deprived for 21–24 hours prior to appetitive training on 1% plain agar. The training vial contains a filter paper with either dried L-glucose or D-glucose that covers the entire wall of the vial. The filter paper was made by applying L-glucose or D-glucose solution (approximately 1 M) and allowing it to dry before use. For control experiment, flies were loaded into a training vial with a filter paper that was soaked in

water and allowed to dry. Conditioning was performed as follows: ~50 starved flies were transferred to the vial containing dried L-glucose with odor A for 2 min. After 2 min in a clean air vial, they were transferred to the vial containing D-glucose with odor B for 2 min. After 3 min in a clean air vial, we transferred the flies into a T maze where they were allowed 2 min to choose between an arm with the odor A and an arm with the odor B. For all experiments, two groups were trained and tested simultaneously. One group was trained with 3-octanol (OCT, 0.1% in mineral oil) paired as the odor paired with L-glucose and 4-methylcyclohexanol (MCH, 0.14% in mineral oil) paired with D-glucose (indicated as "Experiment A" in Fig. S3a), while the other group was trained with MCH as the odor paired with L-glucose and OCT paired with D-glucose (indicated as "Experiment B" in Fig. S3a). Each group tested provides a half performance index: $PI_{1/2}$ = ([number of flies in D-glucose arm] – [number of flies in L-glucose arm]) / (number of flies in both arms). A final PI was calculated by averaging the two $PI_{1/2}$ (Fig. S3a).

Supplementary figure legends

Figure S1. Starvation and food nutrient content promote feeding behavior from long-term FLIC assays

(**a**) Representative single-choice feeding behavior assayed in FLIC. *Canton-S* flies were starved for 12 (upper), 24 (middle) or 36 (lower) hours before the assays. Blue curves showed electrical current signals that represented the intensity of physical contact between flies and liquid food (100 mM D-glucose). As the original report (1), the a.u. higher than 120 (red line) was considered as feeding events (arrows). Asterisks indicate possible "tasting" events. (**b**) Total feeding time during the 1-hour FLIC assays (n=18-34). (**c**) Representative two-choice feeding behavior assayed in FLIC. *Canton-S* Flies were starved for 36 hours before the assays. Colored curves showed electrical current signals that represented the intensity of physical contact between flies and liquid food (blue: 100 mM D-glucose; orange: 100 mM L-glucose). (d) Total feeding time during the 1-hour FLIC assays (n=25). Sample size for each data set was summarized in Table S1. Error bars represent SEM. ns, $P > 0.05$; $*P < 0.05$; $**P < 0.01$; $***P < 0.001$; $****P<0.0001$. Student's t-test was used for pair wise comparisons. One-way ANOVA followed by Bonferroni *post hoc* test was used for comparisons for more than 2 groups.

Figure S2. Starved flies also exhibited preference towards nutritive D-glucose at high concentrations

(**a**) Volume of 500 mM D-/L-glucose consumed in a meal, by *Canton-S* flies starved for 12, 24 and 36 hours (n=24-25). (**b**) Volume of 1 M D-/L-glucose consumed in a meal, by *Canton-S* flies starved for 12, 24 and 36 hours (n=24-26). Sample size for each data set was summarized in Table S1. Error bars represent SEM. ns, P > 0.05; *P < 0.05; **P < 0.01; ***P < 0.001; ****P<0.0001. Two-way ANOVA (and *post hoc* test if applicable) was applied for comparisons with more than one variant.

Figure S3. SLC5A11 is required for associative learning between a nutritious sugar and an odorant in a short time window

(**a**) Schematic illustration of the appetitive olfactory conditioning assay pairing an odor presentation with L-glucose followed by another odor with D-glucose. For details please refer to "Materials and Methods" section. MCH: 0.14% 4-methylcyclohexanol. OCT: 0.1% 3-octanol. (**b**) Preference of flies when given a choice between two indicated odorants after the appetitive olfactory conditioning. Preference index (PI) measures the preference towards the odorants associated with D-glucose. PI=1 means that all flies choose the odorants associated with D-glucose, whereas PI=-1 means that all flies choose the odorants associated with L-glucose. Starved *Canton-S* flies exhibited no preference before the conditioning (left) and significantly enhanced preference towards nutritious D-glucose after training (middle). In contrast, $SLC5A11^{1}$ mutants did not develop a preference even after training (right) (n=14-18). Sample size for each data set was summarized in Table S1. Error bars represent SEM. ns, P > 0.05; *P < 0.05; **P < 0.01; ***P < 0.001; ****P<0.0001. One-way ANOVA followed by Bonferroni *post hoc* test was used for comparisons for more than 2 groups.

Table S1. Sample size for each data set

In this table, the sample size (“n=”) for each experimental data set from all figures and supplementary figures are listed for reference.

Supplementary references

1. Ro J, Harvanek ZM, & Pletcher SD (2014) FLIC: High-Throughput, Continuous Analysis of Feeding Behaviors in Drosophila. *PLoS ONE* 9(6):e101107.
2. Krashes MJ & Waddell S (2008) Rapid Consolidation to a radish and Protein Synthesis-Dependent Long-Term Memory after Single-Session Appetitive Olfactory Conditioning in Drosophila. *The Journal of neuroscience : the official journal of the Society for Neuroscience* 28(12):3103-3113.

Figure S1

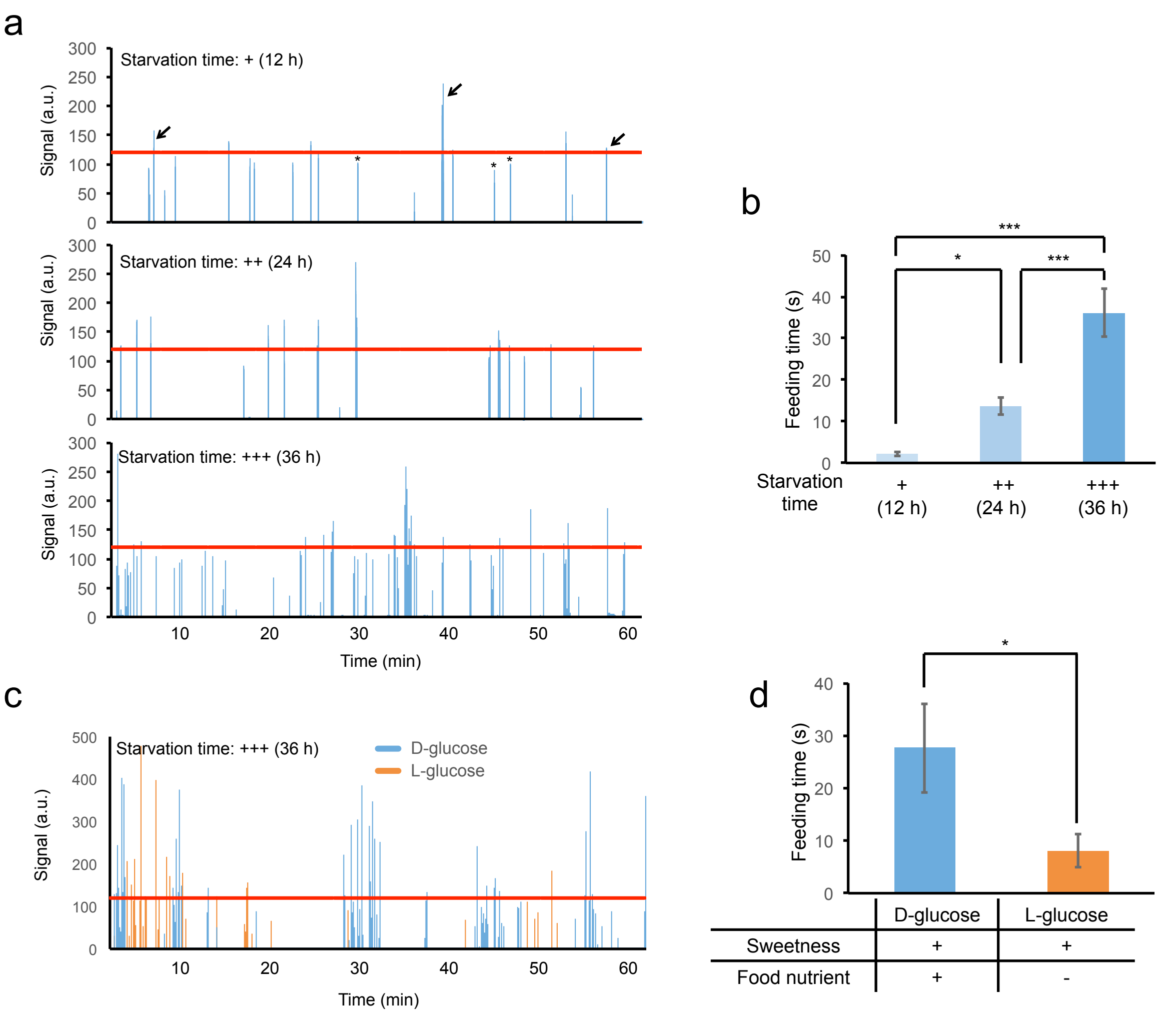
a
Starvation time: + (12 h)
Starvation time: ++ (24 h)
Starvation time: +++ (36 h)
Signal (a.u.)
Time (min)
b
Feeding time (s)
Starvation time
+
(12 h)
++
(24 h)
+++
(36 h)
c
Starvation time: +++ (36 h)
D-glucose
L-glucose
Signal (a.u.)
Time (min)
d
Feeding time (s)
D-glucose
L-glucose
Sweetness
+
+
Food nutrient
+
-

Figure S2

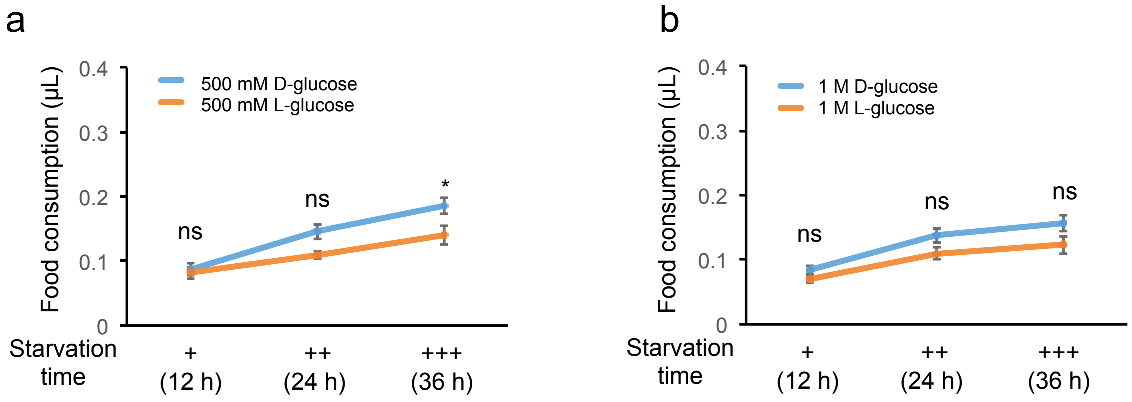
a
500 mM D-glucose
500 mM L-glucose
Food consumption (μL)
0.4
0.3
0.2
0.1
0
ns
ns
*
Starvation time
+ (12 h)
++ (24 h)
+++ (36 h)
b
1 M D-glucose
1 M L-glucose
Food consumption (μL)
0.4
0.3
0.2
0.1
0
ns
ns
ns
Starvation time
+ (12 h)
++ (24 h)
+++ (36 h)

Figure S3

a

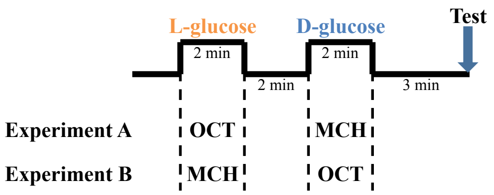
L-glucose
D-glucose
Test
2 min
2 min
2 min
3 min
Experiment A
OCT
MCH
Experiment B
MCH
OCT

b

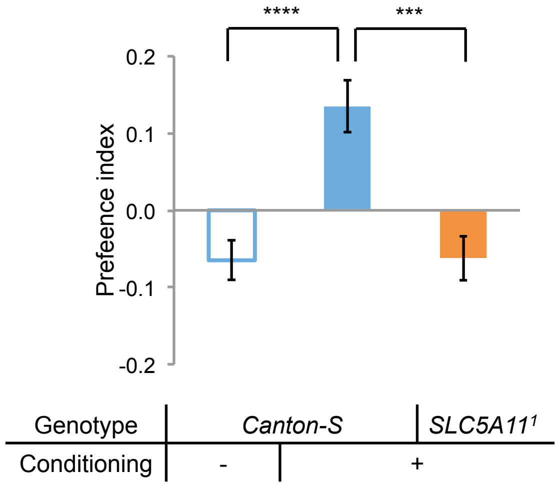
****
***
0.2
0.1
0.0
-0.1
-0.2
Prefeence index
Genotype
Canton-S
SLC5A11[1]
Conditioning
-
+

Supplementary Table 1

Figure 1

(d) n=64

(e)

Feeding bouts	total	1st	2nd	3rd	rest
n	102	37	40	29	29

(f)

Time after a meal	0 min	0.5 min	1 min	5 min	10 min	Meal size
n	20	23	25	22	29	102

Figure 2

(a)

Starvation time	Fed (0 h)	+ (12 h)	++ (24 h)	+++ (36 h)
n	29	26	25	25

(b)

Starvation time	+ (12 h)	+++ (36 h)
n	34	29

(c)

Starvation time	+ (12 h)	+++ (36 h)
n	34	29

(d)

HGN1-GAL4		+	+	-
UAS-Shibire^{ts1}		+	-	+
n	20 °C	29	29	29
	30 °C	28	24	24

(e)

HGN1-GAL4		+	+	-
UAS-TRPA1		+	-	+
n	20 °C	28	25	35
	30 °C	26	21	16

Figure 3

(a)

Starvation time		+ (12 h)	++ (24 h)	+++ (36 h)
n	D-glucose	34	33	36
	L-glucose	33	30	36

(b)

Starvation time		+ (12 h)	++ (24 h)	+++ (36 h)
n	D-glucose	34	33	36
	L-glucose	33	30	36

(c)

Starvation time		+ (12 h)	++ (24 h)	+++ (36 h)
n	D-fructose	24	33	36
	L-fructose	19	26	32

(d)

Starvation time		+ (12 h)	++ (24 h)	+++ (36 h)
n	D-fructose	24	33	36
	L-fructose	19	26	32

(e)

L-glucose (mM)	100	90	50	10	0
D-glucose (mM)	0	10	50	90	100
n	26	22	24	35	25

(f)

Fed with:	D-glucose	L-glucose
Re-stimulated with:	L-glucose	D-glucose
n	26	25

(g)

		First feeding bout	Total bouts
n	D-glucose	24	30
	L-glucose	25	28

(h)

	D-glucose	L-glucose
n	69	68

Figure 4

(a)

Starvation time		+ (12 h)	+++ (36 h)
n	D-glucose	28	30
	L-glucose	25	32

(b)

Starvation time		+ (12 h)	+++ (36 h)
n	D-glucose	24	42
	L-glucose	27	46

(c)

Starvation time		+ (12 h)	+++ (36 h)
n	D-glucose	28	30

	L-glucose	28	30

(d)

Starvation time		+ (12 h)	+++ (36 h)
n	D-fructose	24	36
	L-fructose	19	32

(e)

Starvation time		+ (12 h)	+++ (36 h)
n	D-fructose	27	27
	L-fructose	25	26

(f)

Starvation time		+ (12 h)	+++ (36 h)
n	D-fructose	33	36
	L-fructose	31	34

Supplementary Figure 1

(a)

Starvation time	+ (12 h)	++ (24 h)	+++ (36 h)
n	34	29	18

(d) n=25

Supplementary Figure 2

(a)

Starvation time		+ (12 h)	++ (24 h)	+++ (36 h)
n	D-glucose	23	25	24
	L-glucose	24	24	25

(b)

Starvation time		+ (12 h)	++ (24 h)	+++ (36 h)
n	D-glucose	26	30	25
	L-glucose	25	26	24

Supplementary Figure 3

(b)

Canton-S	Conditioning -	17
	Conditioning +	18
SLC5A11^{1}	Conditioning +	14
